# General Anesthetic Actions on GABA_A_ Receptors

**DOI:** 10.2174/157015910790909502

**Published:** 2010-03

**Authors:** Paul S Garcia, Scott E Kolesky, Andrew Jenkins

**Affiliations:** Departments of Anesthesiology and Pharmacology, Emory University, School of Medicine, Rollins Research Center #5013, 1510 Clifton Rd NE, Atlanta GA, USA

**Keywords:** GABA_A_ Receptor, General Anesthetic, Isoflurane, Desflurane, Sevoflurane, Propofol, Etomidate, Barbiturates.

## Abstract

General anesthetic drugs interact with many receptors in the nervous system, but only a handful of these interactions are critical for producing anesthesia. Over the last 20 years, neuropharmacologists have revealed that one of the most important target sites for general anesthetics is the GABA_A_ receptor. In this review we will discuss what is known about anesthetic – GABA_A_ receptor interactions.

## INTRODUCTION

General anesthetics have been used to relieve pain and suffering in surgical patients for almost 170 years. Prior to their discovery, surgery was a traumatic and barbaric affair, yet today, it is accepted as a routine and essential part of modern medicine. It is curious therefore, that despite the technological advances that have been made in perioperative medicine and surgical techniques, the pharmacologic therapeutics administered by anesthesiologists to render patients unconscious, continue to be used without a precise understanding of how they generate the anesthetized state. 

Fortunately, over the last two decades, the intensive use of the tools of modern molecular pharmacology and neuroscience has enabled investigators to understand better than ever before how anesthetic chemicals can alter the function of the nervous system. In this review, we will carefully define what we mean by “anesthesia” and then we will focus on how the currently used anesthetic agents modulate the function of the GABA_A_ receptor (GABA_A_R), the most abundant fast inhibitory neurotransmitter receptor in the CNS.

At the outset, we would like to emphasize that this review is neither historical nor is it exhaustive. We do not intend to review the interaction of every drug used by anesthesiologists in the Operating Room, past or present, with every critical binding site in the body. The purpose of this review is to summarize some of the key findings that we feel have advanced our understanding of how the currently used general anesthetic drugs interact with their neuronal targets. The history of anesthetic pharmacology is rich and has played a critical role in our understanding of the anesthetized state and the reader is encouraged to read previous reviews if they are interested in reviewing the action of disused anesthetics such as halothane, chloroform or ether. Neither shall we dwell on discarded theories of anesthetic action. It is the fervent view of the authors that general anesthesia is no different from any other pharmacological process: exogenously administered drugs interact with key sites on cellular proteins in the body which results directly in the alteration in the function of these proteins, in the present case, neuronal proteins that control how information is conveyed through the nervous system. 

It is noteworthy that the study of general anesthesia as a whole, has not immediately lent itself to being accessible to traditional pharmacology; anesthesia is a clinical state where a myriad of behavioral endpoints are caused by a group of drugs that bear no physical resemblance to one another. Nevertheless, it appears that GABA_A_Rs are incredibly accommodating and possess several binding sites that interact with many of the currently used general anesthetics. Moreover, these interactions obey many of the basic principles of rational pharmacology; the effects of drug on protein are concentration dependent, they are subject to the “ceiling effect”, they are allosteric in nature, the effects are dependent on the temperature of the preparation, some compounds exhibit stereoselectivity and depending on the anesthetic, they appear to modulate the efficacy and/or the apparent affinity of the receptor for its endogenous ligand. 

In the following sections, we will define what we understand to be “general anesthesia” and how anesthetic drugs interact with native and mutant GABA_A_Rs. 

## WHAT IS GENERAL ANESTHESIA?

When Oliver Wendell Holmes first coined the term *anæsthesia* in 1846, the word, which literally means a lack of sensation, was used to describe a patient who after inhaling ether vapor, underwent surgery without any apparent suffering. Widespread use of the technique and this term followed, describing a state where one or more chemicals were systemically administered to “anesthetize” patients, permitting humane surgery to occur. Arguably one of the greatest breakthroughs in medical history, it is interesting that this essential component of modern medicine has developed without a rational theory consistent with modern pharmacology that accounts for the molecular mechanisms of these drugs in the human body. 

One reason for this is that after more than 160 years of routine clinical use, there is still no consensus on what singularly defines the anesthetized state. During general anesthesia, a myriad of events occur in the body that alter motor responses, cognition, sensation and autonomic control. A patient will go from being anxious, to unconscious and immobile and back to awake and in light of this, there continues to be a debate on which of these effects, or what combination of these effects are the critical determinants of general anesthesia. 

The seminal work by Eger *et al.* [64], set the benchmark in understanding anesthetic actions for almost 50 years. The definition of the Minimum Alveolar Concentration (MAC) places great emphasis on anesthetic induced immobility, as a read-out for sensation, and less emphasis on processes not directly linked to the sensation of acute pain and their associated motor reflexes. Subsequent research has demonstrated, perhaps not unsurprisingly, that the key sites in the nervous system that determine MAC are found in the spinal cord [3] and interestingly, may not be critically dependent on GABA_A_Rs [73]. Nevertheless, although MAC may not have revealed information on the cerebral sites of anesthetic action and anesthetic actions on sensory processing, one of the great benefits of the MAC studies was to define a set of drug concentrations that are appropriate for use in *in vitro* studies on potential molecular targets, a giant leap forward for molecular pharmacologists interested in understanding how these drugs induce anesthesia. 

For many decades after the inception of general anesthesia, through the lack of available drugs, it was usual for only one general anesthetic drug to be used to induce immobility, analgesia, muscle relaxation, amnesia and unconsciousness all at once. It turns out that anesthesia generated in this way was a dangerous practice. The therapeutic index for these early anesthetic drugs was in the low single digits. Modern anesthesia on the other hand makes use of much safer drugs with higher therapeutic indices and is very rarely achieved with a single drug. Anesthesiologists several devices to measure a patient’s vital signs and actively balance cocktails of drug arrays, with the effect of one drug sparing the patient exposure to higher concentrations of another and in some cases, making use of the synergistic combination of some drug pairs [15, 23, 30, 61]. 

Therefore, to recap: under conditions of optimal monitoring, sedation and anesthesia, it is possible for a patient to undergo surgery and have no recall of intra-operative events. With the safer drugs in clinical practice today, we acknowledge that modest movements may persist if neuromuscular blockade is not employed yet the patient is senseless of their surroundings and surgery can be completed without any adverse events. This satisfies the original definition of anesthesia – no sensation accompanied by a successful surgery. Therefore, a review of the current neuropharmacology of general anesthetic drugs should discuss the non-immobilizing sedative drugs as well as general anesthetics. We acknowledge the important role benzodiazepines play in the perioperative setting but would like to point the reader to an accompanying review in this volume that extensively reviews the interaction of benzodiazepines with GABA_A_Rs and the importance of the these interactions for anxiolysis and sedation. 

## THEORY OF GENERAL ANESTHETIC ACTION 

At the cellular level, anesthetics alter the behavior of neurons, by interacting directly with a small number of ion channels. Under normal conditions, these specialized membrane proteins are activated by chemical signals or changes in the membrane environment. Upon activation, channels change the electrical excitability of neurons by controlling the flow of depolarizing (excitatory) or hyperpolarizing (inhibitory) ions across the cell membrane *via* an ion channel that is integral with the receptor that senses the initial signal. *General anesthetics primarily act by either enhancing inhibitory signals or by blocking excitatory signals*. It is important to note that none of the current clinical general anesthetics are selective for a single ion channel. At clinical concentrations, every anesthetic modulates the function of two or more types of channels in the central nervous system. Thus, each different anesthetic agent alters neuronal activity by acting in differing degrees at multiple sites.

## GENERAL ANESTHETICS AND THEIR TARGETS

Currently, there are 5 inhalational and 5 intravenous anesthetics used to induce or maintain general anesthesia: Inhalational: Nitrous Oxide, Isoflurane, Sevoflurane, Desflurane and Xenon. Intravenous: Propofol, Etomidate, Ketamine, Methohexital and Thiopental. These 10 general anesthetic drugs are often accompanied by sedative benzodiazepines: midazolam, diazepam and lorazepam.

Of these 10 general anesthetics, ketamine, nitrous oxide and xenon inhibit ionotropic glutamate receptors, with the strongest effects being seen on the NMDA receptor subtype. These anesthetics also have modest effects on many other receptors, including GABA_A_Rs, but their primary action is the blockade of NMDA receptors and will not be covered further in this review. 

The other 7 general anesthetics and 3 sedatives share a common target and mechanism of action, they all enhance the function of GABA_A_Rs, the most abundant fast inhibitory neurotransmitter receptor in the CNS. These 7 general anesthetics also have a spectrum of modest to strong effects on other ion channels, including glycine receptors, neuronal nicotinic receptors, 5-HT_3_ receptors, glutamate receptors and the two pore potassium channels [1, 17], with each drug differing in its array of effects, but in order to focus on the GABA_A_R effects, the effects on these other ion channels will also not be considered further in this review. 

## THE GABA_A_ RECEPTOR

### Receptor Diversity and Anatomical Expression

Along with the nicotinic acetylcholine receptor (nAChR), the glycine receptor, and the serotonin type 3 (5-hydroxytryptamine type 3) receptor, GABA_A_Rs are members of the Cys-loop superfamily of ligand-gated ion channels. Each of these receptors is formed as a pentameric combination of transmembrane polypeptide subunits. The name of the superfamily comes from the characteristic “cys-loop” found in all subunits, defined by a disulfide bond between two cysteine residues separated by 13 amino acid residues. Neurotransmitter binding sites are located at two or more extracellular interfaces and in the case of GABA_A_Rs, the endogenous ligand binds between the α and β subunits. So far, nineteen genes have been identified for GABA_A_R subunits (α1 – 6, β1 – 3, γ1 – 3, δ, ε, θ, π, ρ1 – 3)[62]. Despite nearly 2.5 million different possible permutations of subunits, only nine different receptor configurations are unequivocally expressed in significant amounts in the brain [53]. If one includes GABA_A_Rs that can be classified as “tentative” or “existence with high probability”, the number of receptor combinations increases to 26 [53]. Preferred subunit combinations are specifically distributed among different brain regions [69] and even demonstrate different subcellular localization [reviewed in 47]. Because each type of GABA_A_R exhibits distinct biophysical and pharmacologic properties, these receptors are capable of diverse influences on regulating synaptic transmission and synaptic integration and since these receptors are expressed in parts of the nervous system which process higher-order brain functions, it is not surprising that GABA_A_R function is central to memory, awareness and consciousness. 

## ANESTHETIC ACTION ON NATIVE RECEPTORS

Anesthetic drugs allosterically modulate GABA_A_Rs and disrupt normal physiologic circuits which require precise timing of GABA-ergic input. There exists strong evidence that GABA_A_Rs are involved in mediating some of the classical components of general anesthesia: hypnosis [2], depression of spinal reflexes [13], and amnesia [43]. The contribution of GABA_A_Rs in mediating immobility and analgesia is less clear (see mutagenesis section). Through experimental investigation it is becoming increasingly clear that not only are the different classical components of general anesthesia mediated by different pathways in the nervous system, but different classes of anesthetics can have differing effects within these pathways. 

For example, specific behavioral effects by pharmacologic therapeutics have been linked to different GABA_A_R assemblies present in different brain regions; sedation produced by benzodiazepines has been associated with the α1 containing GABA_A_Rs [44] and anxiolysis appears to mediated by α2 containing GABA_A_Rs in the limbic system but not the GABA_A_Rs in the reticular activating system; which primarily consists of α3 containing GABA_A_Rs [42]. 

Moreover, manipulation of hippocampal GABA_A_Rs by the selective intrasynaptic GABA_A_R blocker, gabazine, revealed that isoflurane selectively enhances intrasynaptic GABA_A_ mediated currents (associated with α1 –2, β2 –3, and γ2 subunits) in comparison to thiopental which is less selective and augmented both intrasynaptic and extrasynaptic (*via *the α5 subunit) GABA_A_ mediated currents [9]. Not only can anesthetics of different classes be selective in their neurophysiological effects, but so can drugs of the same class, for example, synaptic input (frequency and decay time constant) onto hippocampal interneurons is differentially modulated by sevoflurane versus isoflurane [52]. From this handful of examples, it is clear that the anesthetized state produced by each anesthetic is subtly unique and dependent on each drug’s particular pharmacological characteristics in different brain regions. These microscopic observations are supported by autoradiographical measurements of cerebral blood flow and glucose utilization in anesthetized rodent brains which revealed that specific brain structures could be either hypometabolic or hypermetabolic depending on whether isoflurane or sevoflurane were used [41]. However, the specific effect of each individual volatile anesthetic on specific GABA_A_R subunit assemblies has yet to be systematically examined.

## VOLATILE ANESTHETICS

Nakahiro *et al.* [49] were the first to directly record the enhancement of GABA responses by isoflurane. By patch clamping dorsal root ganglion neurons in primary culture, they were able to enhance the amplitude of currents activated by low concentrations of GABA. Our understanding of the molecular pharmacology of this anesthetic was further enhanced when it was shown that the stereoselectivity for GABA_A_R modulation paralleled its selectivity as an anesthetic [21], strongly implicating the GABA_A_R as a critical target site for isoflurane. However, our understanding of which receptor subunits are critical for general anesthetic action remains incomplete, despite an enormous effort over the last 15 years using the appropriate concentrations of drug (the EC_50_s for general anesthesia translate to the following micromolar concentrations that should be used for *in vitro* experiments at room temperature: 280 µM isoflurane, 330 µM, sevoflurane, 280 µM desflurane [18]) on recombinant receptors in heterologous expression systems [22] and cells that express unique GABA_A_R isoforms. But in general, what appears to be clear is as follows: isoflurane, desflurane and sevoflurane all enhance the amplitude of responses to low concentrations of GABA and prolong the duration of GABA mediated synaptic inhibition. At supraclinical concentrations, they are able to open the receptor’s anion channel in the absence of GABA, a process known as “direct activation”. 

All GABA_A_Rs containing an α subunit are sensitive to at least one volatile general anesthetic drug. The co-transfection of HEK293 cells with β1 subunit cDNAs with either an α1 or an α2 subunit cDNA is sufficient for the expression of GABA_A_ Rs that are sensitive to isoflurane [22]. The addition of the γ subunit does not abolish isoflurane sensitivity, thus we can safely conclude that the binding site(s) for inhaled anesthetics are located, at the very least, within alpha and beta subunits. This is in contrast to homomeric receptors that are constructed from rho subunits and whose responses are inhibited by volatile anesthetics [45]. 

**Fig. (1):**
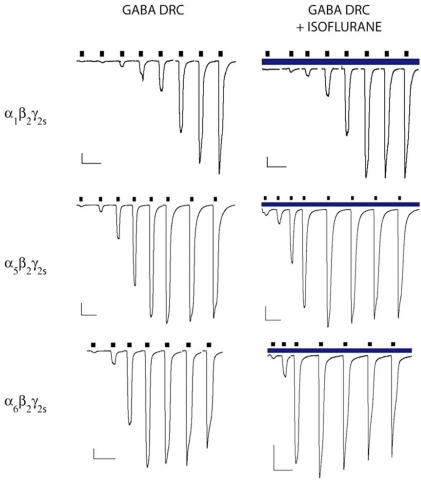
Examples of chloride currents for α_1_β_2_γ_2s_, α_5_β_2_γ_2s_, and α_6_β_2_γ_2s_ GABA_A_ receptors. Left panels show representative whole-cell recordings achieved by applications of increasing concentrations of GABA (black bars). Right panels show paired recordings with the same concentrations of GABA in the presence of 2 MAC isoflurane (blue bar). GABA concentrations (all in µM) for α_1_β_2_γ_2s_ GABA_A_ receptors (0.3, 1, 3, 10, 30, 100, 300, 1000), for α_5_β_2_γ_2s_ GABA_A_ receptors (0.1, 0.3, 1, 3, 10, 30, 100, 300), and for α_6_β_2_γ_2s_ GABA_A_ receptors (0.1, 0.3, 1, 3, 10, 30, 100). Vertical scale bars 100 pA; Horizontal scale bars 10 s. The data indicate that α_6_ containing receptors are more sensitive to low concentrations of anesthetic than α_1_β_2_γ_2s_, or ;_5_β_2_γ_2s_ receptors and that isoflurane is more effective at reducing the apparent affinity of GABA at α_5_ containing receptors than at α_1_β_2_γ_2s_, or α_6_β_2_γ_2s_ GABA_A_ receptors. The methods used to collect these results have been described previously [59].

Research on the effects of volatiles at extrasynaptic GABAARs in thalamic [8] and hippocampal [4] brain slice preparations suggest that the extrasynaptic GABAARs exhibit higher affinities for GABA and exhibit decreased desensitization. At present, there is great interest in the relevance of extrasynaptic receptors in generating the anesthetized state, with many laboratories investigating the importance of the anesthetic enhancement of CSF-activated tonic responses that are associated with extrasynaptic sites (reviewed in [6]). According to our own measurements, CSF contains ~150 nM GABA (A.J & P.S.G., data not shown). The resulting anion shunt is currently thought to be critical to the behavior of neurons in different brain regions. Similarly, the molecular actions of inhaled anesthetics on recombinant subunit combinations (α4—6, and δ- containing GABA_A_Rs) thought to be associated with these extrasynaptic receptors are also being intensively investigated. Recent results in our laboratory using HEK293 cells expressing either α1β2γ2s, α5β2γ2s, or α6β2γ2s GABA_A_Rs have demonstrated that each of these subunit combinations exhibits enhanced GABA sensitivity with isoflurane in a dose-dependent fashion, but the α5- and α6- containing subunit combinations had a heightened GABA sensitivity and the α6β2γ2s combination demonstrated decreased desensitization, together with modest differences in isoflurane induced enhancement (see Table **1** and Fig. **1**). Evidence such as this effectively closes the loop on the hypotheses of extrasynaptic GABA-ergic transmission garnered in brain slice preparations. Our data is consistent with the observation that low, sub-anesthetic concentrations of isoflurane potentiate the Cl^-^ current at α5β3γ2L GABA_A_Rs but not at α1β3γ2L GABAARs in hippocampal neurons [11]. It is important to recognize that dose can play an important role in characterizing the effects of anesthetics on these receptors, either alone or in combination with other anesthetics because drug interactions vary considerably at different time points during surgery and along a response surface [59]. Similarly, we feel that systematic investigations of drug actions on subunit combinations provides a clearer picture as to the effects of those drugs even if some of those subunit combinations have not been identified yet in the mammalian CNS. As it stands today, the literature is lacking a complete survey of the effect of isoflurane, sevoflurane and desflurane on the 26 GABA_A_R populations found in the CNS of a single species, using comparable methodologies, be it the kinetics of ion channel function or shifts of the GABA concentration response. 

Desflurane and sevoflurane are both known to have similar actions on GABA_A_Rs as isoflurane; both drugs increase the apparent affinity of the receptor for GABA. However, much less is known about their relative effects on different GABA_A_ R subunits. Synaptic receptors containing α1, α2, β1 and β2 containing receptors are sensitive to at least one of these drugs, but their relative effects on extrasynaptic receptors has yet to be studied [28, 51, 59]. 

Finally, there has yet to be published an unambiguous demonstration of volatile anesthetic action on GABA_A_Rs at the single channel level. While many studies using fast solution exchange have inferred that volatile anesthetics alter one or more rate constants that govern channel function [31], the drug effects on the lifetimes of specific states remain unknown. 

## INTRAVENOUS ANESTHETICS 

In common with the inhaled anesthetics, the intravenous anesthetics (thiopental, etomidate and propofol) enhance the amplitude of responses to low concentrations of GABA at clinically relevant concentrations (2 µM propofol, 3 µM etomidate, 25 µM thiopental and 10 µM methohexital) and prolong the duration of GABA mediated synaptic inhibition. At supraclinical concentrations, they also directly activate the receptor’s anion channel. 

The interactions between GABA_A_Rs and the intravenous anesthetics are generally thought to occur within, or proximal to, the β subunits. Since the β subunit shows less specific subcellular localization than that of the α subunit, the intravenous anesthetics may not demonstrate rich physiological differences among intra- vs extra-synaptic GABA_A_Rs throughout the brain. Additionally, the distribution of the β subunit in mammalian brain does not share the same clear distinctions as the distribution of the α subunit[40, 69]. By examining in situ hybridization data, one clear trend emerges: the β1 subunit is largely confined to the hippocampus while the β2 and β3 subunits appear more widely distributed. 

### Propofol

The dialkylphenol, propofol (2,6, diisopropylphenol) potentiates GABA responses and directly activates GABA_A_R function [55]. Initially, only the property of direct activation of the GABA receptor by propofol was assumed to be dependent on the β subunit [55, 56] while the modulatory effects were considered to involve other subunits [56]. There is evidence that α, β and γ subunits all contribute to GABA_A_R sensitivity to propofol [20, 32, 39]. In particular, propofol was shown to be less efficacious at β1 containing receptors than at those containing β2 or β3 subunits [57]. The potency and the efficacy of the receptor – drug interaction is dependent on key propofol moieties, most notably, the phenolic hydroxyl group and the number and arrangement of methyl groups at the 2- and 6- positions that flank it [37]. Interestingly this study indicated that the 4-position played little role in modulation. The discovery of the essential pharmacophore for propofol’s action, it has recently led to the development of FOS-propofol, a new anesthetic that releases a propofol molecule after liver metabolism. This provides the anesthesiologist with a water soluble anesthetic that has slower induction kinetics than it’s active metabolite, which may be desirable in some surgical settings [48]. 

Finally, propofol may be actively involved in the recruitment of new subunits to the surface of the neuron; quantitative PCR revealed that during deep anesthesia propofol, increases α4 subunit mRNA occurred in comparison with midazolam, thiopental and isoflurane [60]. 

### Etomidate

The effects of etomidate on GABA and benzodiazepine binding at the GABA_A_R have been known for over 25 years [65]. Like isoflurane, etomidate interacts with the GABA_A_R in a stereospecific manner [67]. Etomidate has smaller effects on receptors containing the β1 subunit [24]. Of all of the clinically used anesthetics, etomidate has the greatest selectivity for the GABA_A_R, and has the fewest relevant interactions with other ion channels in order to generate the anesthetized state. 

### Thiopental

Besides methohexital which has a special role in electroconvulsive therapy [26], thiopental is the only barbiturate routinely used by modern anesthesiologists. Like etomidate, it is known to enhance GABA_A_R function in a stereospecific manner [68]. This is also true for other barbiturate sedatives such as hexabarbital and pentobarbitial. The action of the latter drug has been confirmed at the single channel level, in what is arguably the most detailed study of a general anesthetic modulating the GABA_A_R. Steinbach and Akk [63] demonstrated the complexity of the anesthetic-receptor modulation, but were also able to clearly show that pentobarbital enhanced channel function by stabilizing one of the open states. In common with high concentrations of propofol and etomidate, thiopental can also directly gate GABA_A_Rs. 

Thiopental appears to be selective for either tonic or phasic GABA_A_R activity in the hippocampus [9] suggesting a possible role for the δ subunit in controlling thiopental actions on GABA_A_Rs. This is interesting since the molecular site of action of barbiturates at GABA_A_Rs has long been thought to reside within one of the β subunits. Thus, it would appear that barbiturate actions on GABA_A_Rs are more complex than with other anesthetics [63]. This observation is supported by the fact that thiopental is a more effective agonist than GABA in receptors containing α6 subunits [14]. 

## MUTANT RECEPTORS AND GENETICALLY MANIPULATED ANIMALS

In 1997, Neil Harrison and Adron Harris published the first report of GABA_A_Rs that had been designed to be insensitive to inhaled anesthetics [46]. The rationale behind the ground-breaking work was similar to the successful strategy that had revealed the functional target site of benzodiazepines 4 years earlier [35], taking advantage of the fact that the function of receptors constructed from the ρ1 subunit was not enhanced by inhaled anesthetics [45]. Through a series of receptor chimeras, they identified a pair of transmembrane amino acid substitutions that were sufficient to render α2β1 containing receptors insensitive to enflurane, an inhalational anesthetic (and a chemical isomer of isoflurane). By replacing the α2 residues serine270 and alanine291 with the corresponding residues in the rho1 subunit, an isoleucine and tryptophan respectively, the recombinant receptors were no longer enhanced by the anesthetic. One week later, it was reported that a homologous residue in the β3 subunit was critical for the action of intravenous anesthetics [7]. 

During the following decade, mutations have been introduced into many of the GABA_A_R subunit transmembrane domains, using a variety of rationales, to delineate the general anesthetic target sites in each receptor subunit (see Table **2**). In all cases, the mutation of one or more transmembrane residues has resulted in the impairment or abolition of anesthetic modulation by either isoflurane, desflurane, sevoflurane, propofol or etomidate. Each of these studies concluded that amino acid substitutions alter the molecular environment in the anesthetic binding cavity, presumably by reducing the number of favorable interactions between the receptor and the anesthetic molecule and thus reduced the effect of the anesthetic on enhancing GABA_A_R function. In common with the work with the different subunit combinations, our understanding of the specific residue-anesthetic interactions is also far from complete. 

For the inhaled anesthetics, the majority of the published studies have focused on the interactions of isoflurane with mutant receptors and in comparison, much less is know about the interaction of sevoflurane and desflurane with mutant receptors. That said, where comparisons are possible, it appears that all three inhaled anesthetics behave in qualitatively similar manners, but it is premature to say for certain whether all inhaled anesthetics interact with their binding sites identically. Only a complete survey of all the critical positions in all of the subunits will reveal if inhaled anesthetics preferentially target specific parts of a given receptor population. But at the current time, it is fair to say that these three anesthetics likely form weak bonds with several residues in the four transmembrane domains of GABA_A_R α subunits as their primary interaction with their critical neuronal targets. 

For many years, a slightly clearer picture emerged for the intravenous anesthetics interacting with β subunits, in part due to the relative ease of conducting experiments with non-volatile compounds, the smaller number of β subunits and the smaller number of IV anesthetics. Again, residues in the 4 transmembrane domains appear to be critical in defining the actions of etomidate, propofol and barbiturates. 

It is interesting to note that the mutation of many residues in the transmembrane domains have modest effects on anesthetic modulation of GABA_A_Rs. However, the strongest effects seem to be reserved for homologous positions in transmembrane domains M2, M3 and M4 respectively (see Table **2**) and there is evidence to suggest that this pattern extends across several members of the LGIC superfamily [36]. 

The full potential of these studies was realized in 2003 and again in 2005 when the first reports of the incorporation of some of these mutations into the mouse genome were reported [25, 34]; the mouse lines generated exhibited a selective loss of sensitivity to intravenous and inhaled anesthetics respectively. These animals and their lines that they have inspired [10, 16, 70-72] are now being used extensively to understand the role different GABA_A_R subunits are playing in generating the anesthetized state. 

## CONCLUSIONS

Our current understanding of the molecular mechanisms of general anesthesia will not be complete until we understand how drug action at multiple sites alters the integration of information in the nervous system. Central to this will be an understanding of which circuits in the nervous system are most important for generating sensation and how perturbations at receptors in these circuits generates a state of *anaisthesis*. We have already seen how GABA receptor modulation leads to the sleep-like state associated with anesthesia [50]. Using the knowledge derived from the studies described here, we should learn in the coming years a great deal more about the molecular mechanisms of sleep, sensation, pain and perhaps one day, consciousness.

## Figures and Tables

**Table 1.** 

	Isoflurane 140 µM	Isoflurane 280 µM	Isoflurane 560 µM	Isoflurane 1400 µM
α_1_β_2_γ_2s_	0.35 ± 0.06	0.54 ± 0.06	0.56 ± 0.02	0.58 ± 0.03
α_5_β_2_γ_2s_	0.28 ± 0.05	0.54 ± 0.03	0.57 ± 0.05	0.75 ± 0.03
α_6_β_2_γ_2s_	0.44 ± 0.09	0.57 ± 0.12	n.d.	0.60 ± 0.08

Fractional Effect of Isoflurane on GABA EC50 for α_1_β_2_γ_2s_, α_5_β_2_γ_2s_, and α_6_β_2_γ_2s_GABA_A_ receptors. Values are mean ± SEM and are determinations of at least 4 concentration-response shifts from at least 3 cells, as determined by the fractional change in the effective GABA concentration for 50% of maximal activation (EC50). Isoflurane concentrations are reported as molar concentrations. These concentrations equate to 0.5, 1, 2 and 5 times isoflurane minimum alveolar concentration (MAC). n.d. (not determined).

**Table 2.** 

Transmembrane Domain				
Subunit	M1	M2	M3	M4
α1	[5]	[25]	[33]	[27]
α2	[29]	[46]	[46]	-
α3	-	[58]	[58]	-
β1	[19]	[66]	-	-
β2	[12]	[7]	[38]	[54]

Transmembrane domain mutations in most of the synaptic GABA_A_ receptor subunits reduce modulation by general anesthetics. M1 denotes transmembrane domain number 1. References are from a mixture of species, comprising human, rat and mouse gene products. A dash denotes that to our knowledge, the domain has not yet been mutated to render a significant effect on the effect of a clinically used anesthetic.
